# Effects of Clear-Sky Assimilation of GPM Microwave Imager on the Analysis and Forecast of Typhoon “Chan-Hom”

**DOI:** 10.3390/s20092674

**Published:** 2020-05-08

**Authors:** Dongmei Xu, Aiqing Shu, Feifei Shen

**Affiliations:** 1 Key Laboratory of Meteorological Disaster, Ministry of Education (KLME)/Joint International Research Laboratory of Climate and Environment Change (ILCEC)/Collaborative Innovation Center on Forecast and Evaluation of Meteorological Disasters (CIC-FEMD), Nanjing University of Information Science & Technology, Nanjing 210044, China; dmxu@nuist.edu.cn (D.X.); aqshu@nuist.edu.cn (A.S.); 2 Heavy Rain and Drought-Flood Disasters in Plateau and Basin Key Laboratory of Sichuan Province, Chengdu 610225, China

**Keywords:** WRF model, GMI radiance data, 3DVar, typhoon forecast

## Abstract

The module of assimilating a new GMI (GPM Microwave Imager) satellite detector was built in the framework of the Weather Research and Forecasting Model (WRF) and its three-dimensional variational (3DVar) data assimilation system (WRFDA). Typhoon “Chan-Hom” in the 2015 Pacific typhoon season was selected to verify the effectivity of the GMI clear-sky assimilation. The results show that, after assimilating the GMI radiance data, the background information in the model is modified positively when compared with the experiment without any assimilation and the one with assimilation of the conventional data. The obvious warm core structure of the typhoon, the modified geopotential height field, and the intensified circulation of the typhoon are favorable for the northwest twist of the typhoon, thus contributing to a better track forecast with a maximum error below 160 km in the 48-h deterministic forecast.

## 1. Introduction

Since the late 20th century, because of the launch of many new meteorological satellites and the rapid development of advanced data assimilation (DA) methods, satellite data has become one of the main sources of observation data which is used in numerical weather prediction (NWP). For the satellite radiance data, the simulated radiance can be obtained through the rapid radiation transmission model when the atmospheric state, satellite scanning model and fixed surface attributes are given, and then the background field of numerical simulation can be modified according to the differences between the simulated radiance and the observed one, leading to an optimal analysis of the current atmospheric state [1]. A large number of previous studies show that, in global [2,3,4,5,6,7,8,9] and regional models [10,11,12,13,14,15,16,17], direct assimilation of satellite radiance data can improve the accuracy of NWP.

Until now, the direct assimilation of satellite radiance data has been recognized as an effective way to reduce the errors of NWP. However, there is relatively little conventional observation data over the broad ocean, which brings some difficulties and challenges to the analysis and prediction of tropical cyclones (TCs). In the last few decades, many experts and researchers have been committed to the direct assimilation of satellite observation data to effectively improve the prediction of the track and intensity of TCs [18,19,20,21,22,23,24,25,26]. The Global Precipitation Measurement (GPM) [27], whose main satellite was launched on 28 February 2014, with joint participation from the National Aeronautics and Space Administration (NASA) and Japan Aerospace Exploration Agency (JAXA), aims to monitor global precipitation, thus effectively improving the accuracy of weather forecasts. It is equipped with a dual-frequency precipitation radar (DPR) and a GPM microwave imager (GMI). The GMI not only inherits nine channels from its previous generational imager, the TRMM microwave mmager (TMI) devised by Tropical Rainfall Mission (TRMM) [28] to monitor heavy rain to light rain, but also adds four high-frequency channels to effectively monitor snowfall. In addition, the spatial resolution of the GMI is twice of that of the TMI, which is higher than any other former satellite precipitation product.

By now, some researches about GMI DA have been done to improve typhoon prediction and encouraging results have been achieved. Based on two Atlantic hurricanes in 2015 and 2016, Pu et al. (2018) [29] use the Hurricane Weather Research and Forecasting (HWRF) Model and the Gridpoint Statistical Interpolation (GSI) assimilation system to make assessments on the clear-sky assimilation of GMI brightness temperature and make a comparison of other prevailing microwave sounders like the advanced microwave sounding unit-A (AMSU-A), the advanced technology microwave sounde (ATMS), the microwave humidity sounding (MHS), etc., they found that clear-sky assimilation of GMI radiance is able to positively improve both track and intensity forecasts. Since most of these researches focus mainly on TCs over the Atlantic Ocean rather than the Western Pacific Ocean, the direct assimilation of the new generational GMI radiance data in a limited regional area, especially in East Asia, has not been carried out. Furthermore, the effectivity of the clear-sky three-dimensional variational (3DVar) method for GMI DA in the WRFDA system has not been verified and there is still a lot of work to be done. Therefore, this study intends to adopt the new generational Weather Research and Forecasting Model (WRF) and its three-dimensional variational data assimilation system (3DVAR) to address the influence of the direct assimilation of GMI radiance data on the analysis and prediction of Typhoon “Chan-Hom” in July, 2015.

## 2. Observation Data and Assimilation System

### 2.1. GMI Microwave Imager Data

The GMI Microwave Imager is a passive radiometer jointly devised by the National Aeronautics and Space Administration (NASA) and Japan Aerospace Exploration Agency (JAXA) [27]. It is mainly used to measure cloud precipitation information. The GMI adopts conical scanning to the ground, with a scanning width of 885 km and an elliptical field of view. In addition, the GMI antenna diameter is increased from 61 cm to 1.2 m, so that higher spatial resolution can be obtained. In terms of detection frequency bands, the GMI includes eight frequencies and 13 channels including low frequency bands ranging from 10 GHz to 89 GHz and high frequency bands ranging from 166 GHz to 183 GHz in Table 1. It should be mentioned that the charge-induced effects in water droplets may be the additional source of anomalies at microwave frequencies contributing to the model thermal fields [30]. The frequencies of channels 1 to 9 (10 GHz to 89 GHz) are similar to those of their predecessor TMI microwave imagers. Among them, the 10 GHz low-frequency channel is only sensitive to liquid precipitation, mainly monitoring the precipitation and water vapor distribution in the lower troposphere. The 183 GHz high-frequency channel is used for deep detection of the atmosphere, which is sensitive to the ice phase condensation, so the scattering signal of small ice particles can be detected. In addition, light rain and snowfall are monitored by the other four high frequency channels. The GMI has not only the ability to detect precipitation, but also the ability to extend the coverage to midlatitude areas.

### 2.2. The WRFDA Assimilation System

The Weather Research and Forecasting model data assimilation system (WRFDA) is developed and maintained by National Centers for Atmospheric Research (NCAR), which is an affiliated part of the mesoscale numerical model WRF (Weather Research and Forecasting model). It consists of the three-dimensional variation (3DVAR) and the four-dimensional variation (4DVAR) data assimilation systems, and hybrid assimilation [31]. In our research, the WRFDA assimilation system is expanded and a module designed for GMI Microwave Imager data assimilation has been built independently. In order to test the new assimilation module, this study adopts the traditional 3DVAR assimilation method, which provides the best analysis field through the iterative minimization of cost function J(x):

(1) $$J\left(x\right)=\frac{1}{2}{\left(x-x_{b}\right)}^{T}B^{-1}\left(x-x_{b}\right)+\frac{1}{2}[\left(H\left(x\right)-y_{0}{)}^{T}O^{-1}\left(H\left(x\right)-y_{0}\right)\right]$$

where, x is the analysis variable (analysis vector); $x_{b}$ is the background field, generally the model forecast field; B is the background error covariance matrix; $y_{0}$ is the observation field; O is the observation error covariance matrix; $B^{-1}$ is the inverse matrix of B; $O^{-1}$ is the inverse matrix of O; y=H(x) is the observation equivalent of the analysis variable [32,33]. By calculating the minimum value of the cost function, the estimation of the real atmospheric state at the analyzed time is obtained. Thus, the optimal analysis $x_{a}$ field is found.

### 2.3. The Build of GMI Assimilation Module

In the current WRFDA assimilation system, the assimilation part of GMI Microwave Imager data does not exist. Therefore, it is necessary to build the assimilation module for GMI Microwave Imager data. In this study, the Community Radiative Transfer Model (CRTM) is employed to simulate GMI radiance data. On the one hand, the scanning time, the earth incident angle and azimuth angle, the sun azimuth angle and altitude angle, the longitude and latitude information and the brightness temperature value are read from GMI level 1 data in HDF5 (Hierarchical Data Format 5) format to provide input information for the simulation and calculation of brightness temperature of the background field. On the other hand, the cloud liquid water path (CLWP) value in cloud read from the level 2 file is used for quality control of observation data.

In the assimilation system, the quality control of observation data is very important. In order to ensure the correctness of the experiment results, based on some former quality control methods for other satellite observation data, a special quality control scheme suitable for GMI Microwave Imager data is also considered, and the final quality control methods for GMI are included as follows:

(1) When reading GMI observation data, remove abnormal observation values, such as those less than 50 K and more than 550 K, following Shen F. et al. (2015) [34], Yang C. et al. (2016) [24] and Zou X. et al. (2013) [35] to exclude the invalid brightness temperature.
(2) Considering the complex underlying surface of the terrain, only the observations over sea are assimilated, and the observations on the land and the observations with comparatively complex types of sea surface are excluded. Those complex types refer to the mixed predominately sea, mixed predominately sea ice, mixed predominately land, or mixed predominately snow marked by the WRF forecast model.
(3) According to the differences between the observations and the background simulated brightness temperature, the observations beyond a specific threshold (Table 2) are eliminated.
(4) The observations which are larger than 3$\sigma_{0}$ after the bias corrections are eliminated. $\sigma_{0}$ is the standard deviation of observation (Table 2).
(5) The observations of cloud liquid water path (CLWP) exceeding the threshold value (Table 2) are eliminated. The threshold value is set according to the same frequency band of Chun Yang et al. (2016) [24].
(6) Channel 8 and channel 9 (89.0 GHz) are sensitive to convective precipitation because channel 1 and channel 2 (10 GHz) are greatly affected by surface emissivity. The study by Yang et al. (2016) [24] in terms of selecting channels of AMSR2 is used as a reference. Thus, only the observation data of channels 5, 6 and 7 with similar wavelengths with AMSR2 are chosen in this study.

In this study, GMI Microwave Imager data from 9 July 2015 to 11 July 2015 are used to make statistics of observation errors. According to the differences between the observed brightness temperature and the simulated clear-sky brightness temperature, the standard deviation is calculated as the observation error [24]. It should be pointed out that there are some systematic errors in satellite observation data. Thus, the errors need to be corrected before GMI observation data assimilation. Usually, the linear combination of some predictors is used to express the radiance deviation [36]:

(2) $$\tilde{H}x,\beta =H\left(x\right)+\beta_{0}+\overset{N_{P}}{\underset{i=1}{\sum }}\beta_{i}p_{i}$$

There are three terms on the right side of the equation: The first item H(x) is the initial observation operator (before the bias correction) of GMI DA, which is built in this study in the WRFDA system, x is the model state vector; the second item $\beta_{0}$ is the constant part of the total deviation; in the third item, $p_{i}$ and $\beta_{i}$ are the *i*-th prediction factor and deviation correction coefficient. To the left of the equation, $\tilde{H}\left(x,\beta \right)$ is the modified observation operator. The deviation correction coefficient β is usually assumed to be independent within the channel, and can be calculated through the off-line variational minimization algorithm. This method is called variational bias correction (VarBC) [37]. Bias between observed and simulated brightness temperatures are usually functions of some model variables along with their coefficients. Those model variables are called prediction factors (Liu et al., 2012 [11]; Li et al., 2019 [36]). With the coefficients that are estimated offline initially, the bias can be predicted and corrected. In our research, the WRFDA off-line calculation model of VarBC is used, and GMI observation from 9 July 2015 to 11 July 2015 is used to obtain the deviation correction coefficient at the initial time of GMI assimilation.

## 3. Case Introduction and Experimental Design

### 3.1. Typhoon Introduction

**Figure 1:**
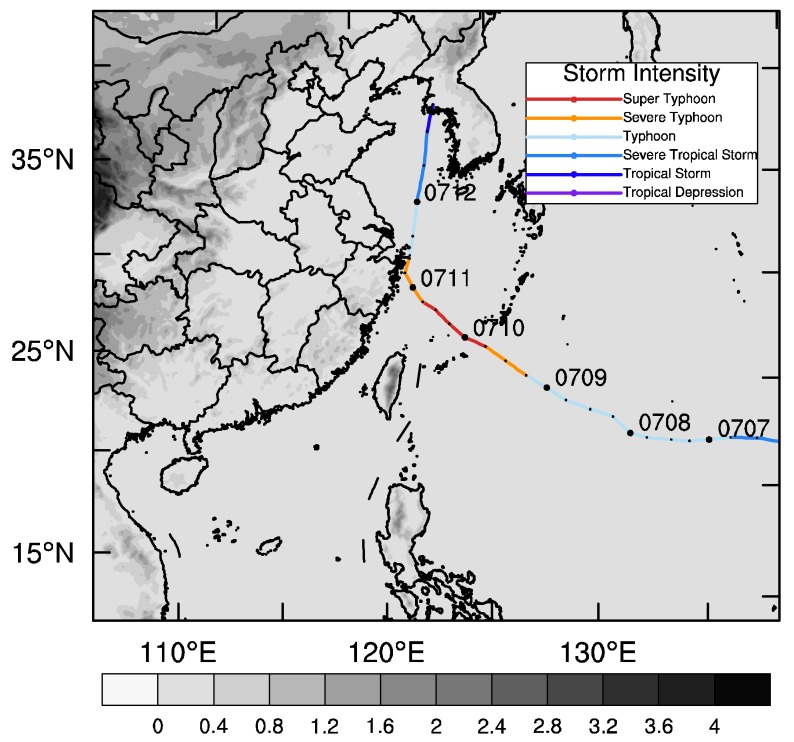
Model domain with terrain height (shaded, unit: km) and the track of Typhoon “Chan-Hom”.

Chan-Hom was formed near the east ocean of the Philippines on 30 June 2015 with a maximum wind of 18 m/s and a minimum pressure of 995 hPa in its core. Then, it was upgraded to a severe tropical storm on 2 July 2015 and a Typhoon on 3 July 2015 by the Japan Meteorological Agency (JMA). Forced by the subtropical anticyclone, it moved northwest and was strengthened gradually. It approached the coast of Fujian and Zhejiang Province in China on 10 July 2015 and landed in Zhejiang Province on the following day. During this process, it went through a rapid intensification to a super typhoon. Afterwards, it reentered the sea, moving northwest by north, and was weakened continuously due to friction and the blocking of sufficient heat and moisture from the tropics. On the night of 12 July it landed again at the Korean Peninsula. After landing, its strength continued to be weakened and its number was stopped. Its track, observed by the China Meteorological Administration (CMA) [38], is partially shown in Figure 1. Because of its long lifespan and high strength, the economic losses were up to RMB 9.8 billion and heavy casualties occurred.

### 3.2. Model Configuration and Experimental Design

In this study, the Advanced Research WRF (ARW) version of WRF V4.0 is employed as the forecast model. The center of the domain is 27° N, 124° E, with a horizontal resolution of 9 km, which is shown in Figure 1. The topmost pressure layer of the model is 10 hPa, and the number of vertical layers is 57. The initial and boundary conditions are generated by 1° × 1° horizontal resolution reanalysis data provided by the National Centers for Environmental Prediction (NCEP). Some parameterization schemes are used in our experiments including the WSM6 (WRF Single-Moment 6-class) scheme [39], the Kain–Fritsch cumulus parameterization scheme [40], the YSU (Yonsei University) boundary layer scheme [41], the Dudhia scheme, and the RRTM (Rapid Radiative Transfer Model) scheme and Noah Land surface model, for short wave radiation and long wave radiation, respectively [42].

**Figure 2:**
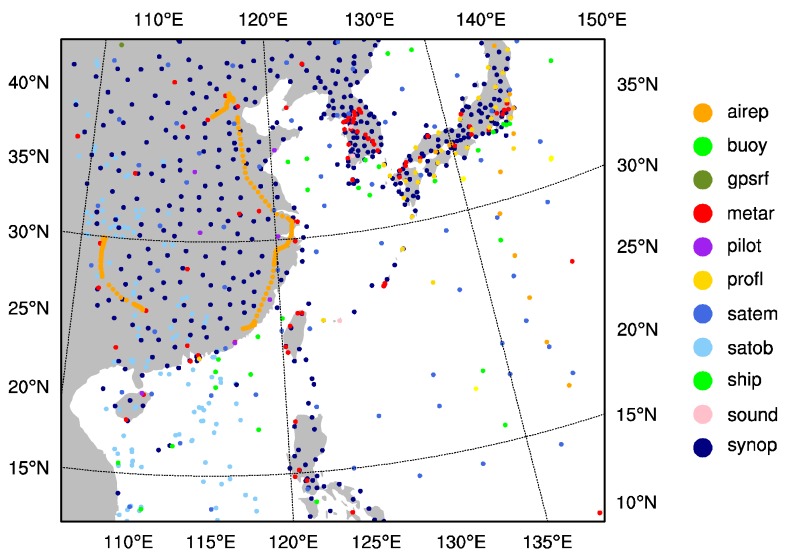
The distribution of GTS observations.

Three steps are performed in this study. Firstly, a 6-h spin-up forecast started at 1200 UTC on 9 July 2015 is performed. Secondly, the forecast field at 1800 UTC on 9 July 2015 is used as the background or first guess field for assimilation. Finally, a 48-h deterministic forecast is carried out. The experiments are classified into three groups. The first experiment is the control experiment (CTNL), which is a pure simulation without any assimilation. The second experiment is the assimilation of conventional observation data from the Global Telecommunications System (GTS_DA), which only assimilates the GFS (global telecommunications system) conventional observation data including the data of aircraft, sounding, ships, ground stations, satellite cloud wind, etc. Figure 2 illustrates the distribution of GTS observation data in the simulation domain at 1800 UTC on 9 July 2015. Based on the second experiment, the third experiment further assimilates the GMI Microwave Imager data under clear-sky conditions (GMI_DA). It is worth mentioning that in order to avoid the potential correlation between adjacent observations, the GMI Microwave Imager data are thinned by 36 km.

**Figure 3:**
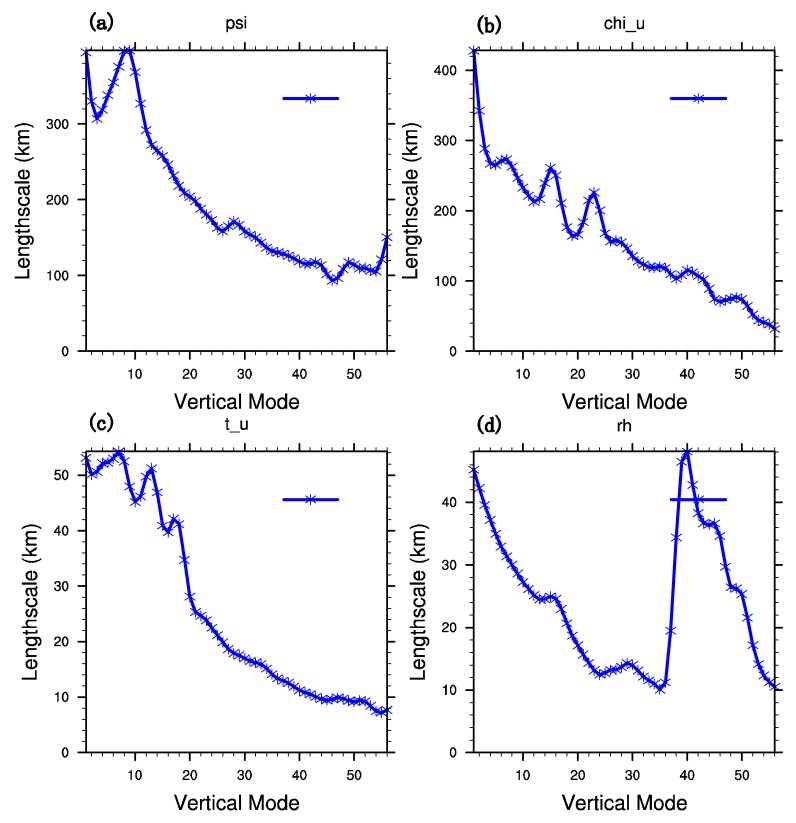
Horizontal length scale of control variables (**a**) ψ, (**b**) χ, (**c**) nonequilibrium temperature, and (**d**) relative humidity.

The background error covariance for assimilation is acquired by the NMC (National Meteorological Center) method [43]. The detailed method is making 24-h and 12-h forecasts for 00 UTC and 12 UTC from 1 July to 30 July 2015, respectively. Then, the difference between 24-h and 12-h forecasts at the same starting time is viewed as the approximation of forecast error. In the framework of WRFDA, there are mainly two schemes to calculate the background error covariance matrix B. The one is the CV5 scheme with stream function, the unbalanced velocity potential, the unbalanced temperature, the unbalanced surface pressure, and the pseudo relative humidity as the control variables. Another one is the CV7 scheme with the zonal velocity, the meridional velocity, the temperature, the surface pressure, and the pseudo relative humidity as the control variables. In this study, the CV5 scheme is adopted, which is more commonly used. To get the statistical background error covariance, the balance relationship between the variables is further improved by using the non-equilibrium quantity between the flow function and the background error covariance. By the transformation of control variables in the matrix of background field error covariance, the characteristics of background field error covariance of two dynamic control variables are analyzed. The vertical transformation of the matrix projects the errors of the control variables onto the orthogonal modes in the vertical direction by empirical orthogonal function (EOF), while the horizontal transformation of the matrix uses recursive filter to get model horizontal correlation of individual control variables. Length scale is an important parameter of horizontal transformation by determining the coefficient of recursive filter. Thus, its value can reflect the sphere of influence when observational data are assimilated. Figure 3 is the variation of the length scale of the control variables with different vertical modes. The maximum length scales of the two dynamic control variables—stream function and unbalanced potential function—are around 400 km, which are much larger than that of unbalanced temperature and relative humidity with a maximum of around 50 km, leading the dynamic control variables to bring broader adjustments to the background field. Generally, the length scales of all the control variables decrease with the vertical mode.

## 4. Results

### 4.1. Simulation of the GMI Observation

**Figure 4:**
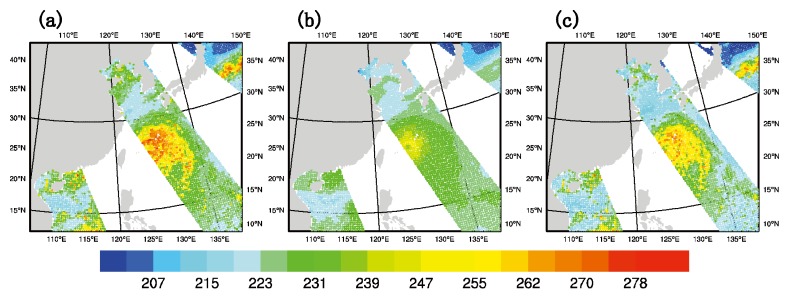
The brightness temperature (unit: K) of channel 6 of (**a**) the observation, (**b**) the background simulation, and (**c**) the analyzed field after data assimilation (DA) at 1800 UTC on 9 July 2015.

To prove the effectiveness of GMI DA, channel 6 is listed to verify the simulation results. The results for the other two assimilated channels yield similar results (not shown). Figure 4 shows the observed brightness temperature, the simulated background brightness temperature, and the simulated analyzed brightness temperature at 1800 UTC on 9 July 2015. Here it should be pointed out that analyzed brightness temperature is simulated with the analyses as the input for the radiative transfer model. Admittedly, due to the effect of charge-induced effects in water droplets, the anomalies at the microwave frequencies may contribute to the improvement in the thermal field [30]. It can be inferred from Figure 4a that there are three scanning tracks within the assimilation window. The middle track covers the east part of typhoon “Chan-Hom” and a clear spiral cloud band can be seen. Therefore, this time is chosen to further address the effect of GMI DA on the analysis and forecast of typhoon “Chan-Hom”. Figure 4b shows the simulated background brightness temperature of the model, which is acquired by a 6-h spin-up prediction. It can be seen that there are three scanning tracks in the whole experimental domain and the spiral cloud band of the typhoon is not vivid enough. In the background field, near the core of typhoon “Chan-Hom”, the overall brightness temperature is weaker than the observed one. Figure 4c is the simulated analyzed brightness temperature. It can be found that the simulated brightness temperature near the core of the typhoon is closer to the observed brightness temperature than in Figure 4b, which shows that the analyzed field is closer to the observed field after assimilating GMI radiance data.

**Figure 5:**
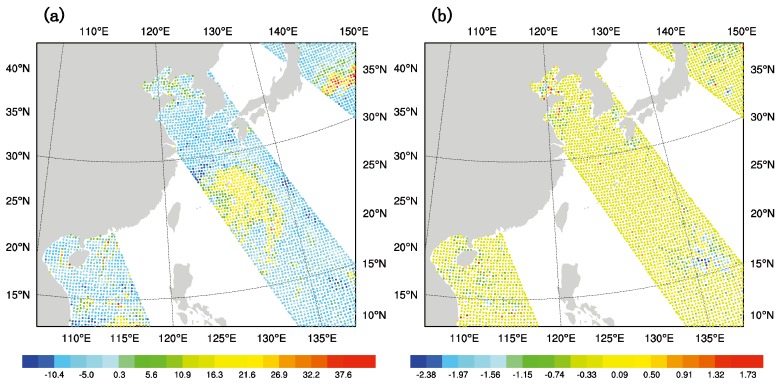
(**a**) OMB (unit: K), and (**b**) OMA (unit: K) of channel 6 after bias correction at 1800 UTC on 9 July 2015.

Figure 5a,b are the observed brightness temperature minus the background brightness temperature (OMB) after the bias correction, and the observed brightness temperature minus the analyzed brightness temperature (OMA) after the bias correction at 1800 UTC on 7 July 2015. There are three scanning tracks in the simulation domain and the typhoon spiral cloud band is covered by the middle track. It can be seen from Figure 5a that most parts near the core of the typhoon have a positive value greater than 10 K and this means the simulated brightness temperature of the background field is lower than the observed brightness temperature. While in Figure 5b, the value of most parts covered by scanning tracks is nearly 0 K, which denotes that the overall difference between the analyzed and the observed brightness temperature decrease after GMI DA. In addition, the brightness temperature error of most scanning points tends to be less than 0.1 K, matching well with the observation.

### 4.2. Bias Correction

**Figure 6:**
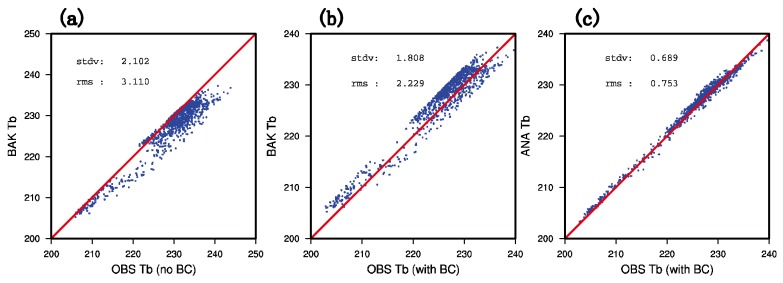
The scatter diagram of the observed brightness temperature (unit: K) and the simulated brightness temperature (unit: K) (**a**) before DA without bias correction, (**b**) before DA with bias correction, and (**c**) after DA with bias correction.

Figure 6a,b show the simulated and observed brightness temperature scatters before and after the bias correction. The abscissa and the ordinate represent the observed brightness temperature value and the simulated brightness temperature value by the CRTM (Community Radiative Transfer Model) observation operator. It can be inferred from the diagram that before the bias correction, the simulated brightness temperature of the background field and the observed brightness temperature have a good correspondence in different brightness temperature ranges. It is noted that most scattered points are below the diagonal, where the observed brightness temperature is equal to the background simulated brightness temperature. This indicates that the simulated brightness temperature of the background is lower than the observed brightness temperature. However, after the bias correction, it can be seen from Figure 6b that the observed brightness temperature and the simulated background brightness temperature are evenly distributed on both sides of the diagonal. Besides, the RMS (root mean square) error is reduced from 3.110 K to 2.229 K with the bias correction. Figure 6c shows that after GMI assimilation, the scattered points are more closely and evenly distributed on both sides of the diagonal. In addition, the RMS error is also significantly reduced from 2.229 K to 0.753 K compared with Figure 6b (before assimilation).

### 4.3. The Impacts on the Analysis

#### 4.3.1. Temperature Increment

**Figure 7:**
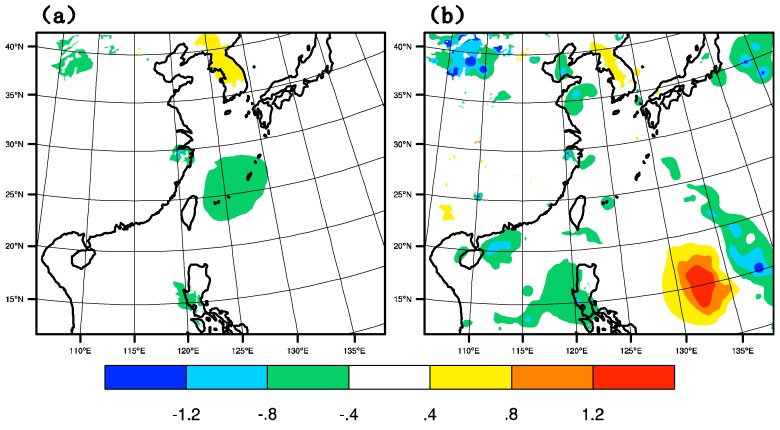
Temperature increment (unit: K) of (**a**) GTS_DA, and (**b**) GMI_DA at 1800 UTC on 9 July 2015.

The temperature increment of GTS_DA and GMI_DA are shown in Figure 7 at 1800 UTC on 9 July 2015 when the location of the typhoon is 25.1° N, 126.6° E. In GTS_DA, near the core of the typhoon, there is a negative temperature increment, which is inconducive to the maintenance of the typhoon. However, in GMI_DA, the sphere of the negative increment is greatly reduced, resulting in a relatively positive increment. Probably due to the theory of charge-induced effects in water droplets, anomalies at microwave frequencies are able to contribute to the model thermal fields [30]. This positive increment is favorable to the warm core structure of the typhoon and the sustainment of typhoon intensity thermodynamically.

#### 4.3.2. Geopotential Height Increment

**Figure 8:**
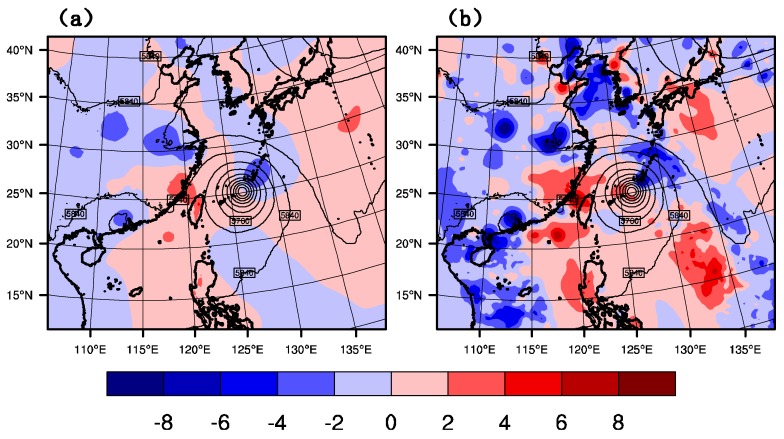
Geopotential height field (unit: gpm) and its increment field at 500 hPa of (**a**) GTS_DA, and (**b**) GMI_DA at 1800 UTC on 9 July 2015.

Figure 8 shows the geopotential height field and its increment field at 500 hPa for GTS_DA and GMI_DA at 1800 UTC on 9 July 2015. Although little difference observed in the two typhoon centers simulated by GTS_DA and GMI_DA, there are big differences in their increment geopotential height fields. After GTS_DA, the northeast part of the typhoon has a negative increment, while the increment in the southwest is a positive value. This height increment can generate a pressure gradient force, which makes the typhoon move northeast compared with CTNL. However, after GMI_DA, the area and the strength of the northeast negative increment and the southwest positive increment are enlarged. In the following section, it can be proved that the correction by GMI_DA on the initial field is more effective than that by GTS_DA, especially in the forecast of typhoon track.

#### 4.3.3. Low-Layer Flow

**Figure 9:**
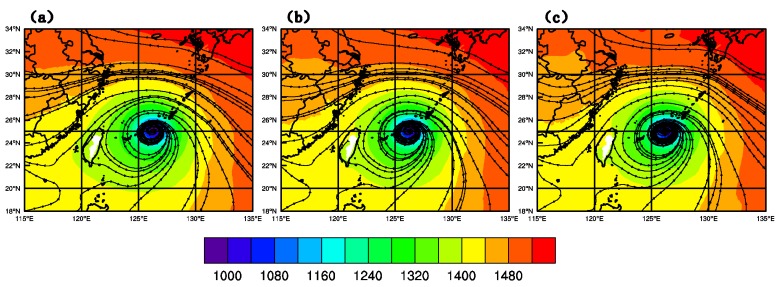
Geopotential height (unit: gpm) and streamlines at 850 hPa of (**a**) CTNL, (**b**) GTS_DA, and (**c**) GMI_DA at 1800 UTC on 9 July 2015.

Figure 9 shows the geopotential height and streamline fields at 850 hPa of CTNL, GTS_DA, and GMI_DA, respectively, at 1800 UTC on 9 July 2015. On the east of the typhoon core, the thickness of streamlines of GTS_DA and GMI_DA is more intensive than CTNL, and the typhoon core of GMI_DA with overlapped lines extends more to the east than in the other two experiments, denoting that the typhoon is intensified after GMI DA.

#### 4.3.4. Steering Current

**Figure 10:**
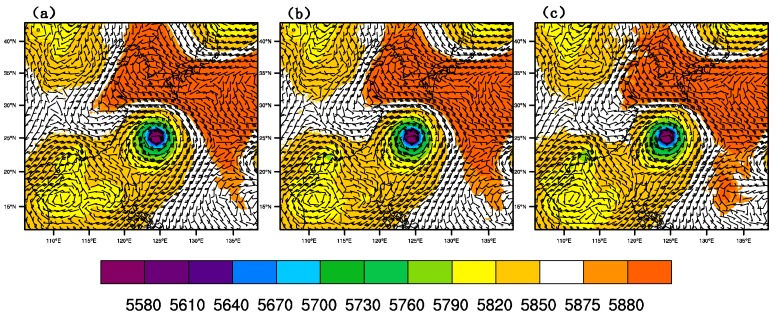
Geopotential height (unit: gpm) and wind field (unit: m/s) at 500 hPa of (**a**) CTNL, (**b**) GTS_DA, and (**c**) GMI_DA.

Figure 10 shows the wind field and geopotential height at 500 hPa of CTNL, GTS_DA, and GMI_DA, respectively, at 1800 UTC on 9 July 2015. For CTNL, the wind speed near the outer side, especially the northeast side, of the typhoon core is weaker than that of GTS_DA, and that of GMI_DA has the strongest wind field. Meanwhile, near the 588 dagpm in the northeast position of the typhoon, the southeast wind component of GMI_DA is larger than that in the CTNL and GTS_DA. It denotes that GMI_DA is able to strengthen typhoon intensity. The relatively larger southeast flow is conductive to driving the typhoon towards the northwest.

### 4.4. Typhoon Track

**Figure 11:**
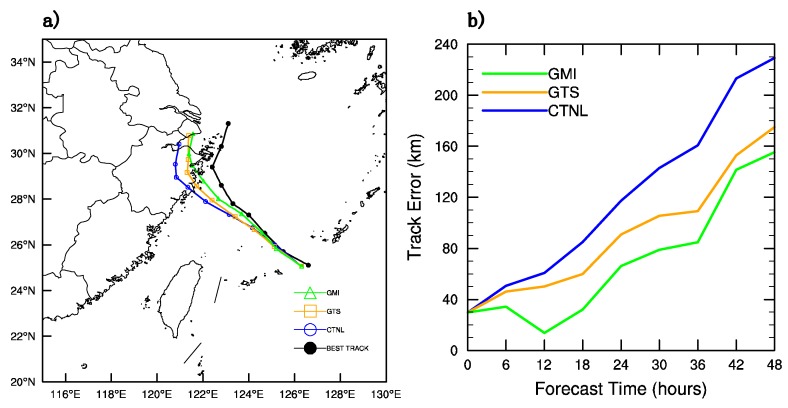
(**a**) the track of three experiments, and (**b**) their error (unit: km) over forecast time.

Further estimation of the influence of the GMI radiance DA on the forecast of Typhoon “Chan-Hom” is performed by a 48-h deterministic forecast started at 1800 UTC on 9 July 2015. Figure 11a shows the forecast track of Typhoon “Chan-Hom”. In the figure, “GMI”, “GTS”, “CTNL” and “BEST TRACK” represent the assimilation of GMI together with GTS in clear-sky, the assimilation of GTS, the control experiment without assimilation and the best typhoon observation data from CMA, respectively. Generally speaking, all of the three forecasting methods are able to describe the tendency of “BEST TRACK” and they all have a north twist when the model is integrated 36 h later. In the first 6 h, the tracks of the three forecasting methods differ very little and both GMI_DA and GTS_DA have an obvious advantage over CTNL in the following 42 h. It can be judged preliminarily that GMI_DA has the least error, followed by GTS_DA, and the CNTL has the largest error. For a quantitative comparison, the error of the three forecasting methods over time is shown in Figure 11b. It is remarkable that there is an initial forecast error of about 30 km due to the 6-h spin-up. In the first 18 h, the forecast error of GTS_DA and CTNL go through a slow increase, but that of GMI_DA decreases from 6 h to 12 h and it remains below 40 km. The track errors from the three experiments increase with the forecast leading time. It can be found that GMI_DA has the best effect since there is consistent reduction of the track error with the largest error less than 160 km compared with those from “GTS” and “CNTL”.

## 5. Conclusions

In this study, the interface of GMI DA was integrated in the WRFDA system and three experiments were performed to address the influence of the clear-sky GMI radiance DA on the analysis and forecast of Typhoon “Chan-Hom” (2015). The following are the main results from these experiments:

(1) The clear-sky assimilation of the new GMI radiance data can capture the shape and structure of Typhoon “Chan-Hom” well. The deviation correction coefficient and quality control obtained by the off-line model statistics of VarBC of WRFDA can improve the assimilation effect of the experiment, reduce the standard deviation of observation residual after 3DVar, and ensure the positive effect of GMI DA on typhoon analysis and forecast.
(2) Compared with GTS_DA, it is found that GMI_DA experiment can effectively increase the warm core structure of the typhoon and modify the geopotential height field in the background field of the model. The circulation of the typhoon is also intensified with GMI_DA, which can contribute to the northwest twist of the typhoon.
(3) In the 48-h deterministic forecast, the track error of the typhoon predicted by the GMI_DA experiment is the smallest, with a maximum value below 160 km, when compared with GTS_DA and CTNL.

The results of this study show that the clear-sky assimilation of GMI radiance data has great potential to improve the typhoon track forecast. The case study of Typhoon “Chan-Hom” also indicates that GMI DA can provide positive effects on typhoon initialization and prediction. This study is a preliminary trial and only a single clear-sky assimilation using the 3DVar method is conducted. Besides, just one typhoon case is chosen for study. Therefore, future work will focus on more typhoon cases and other superior DA methods such as the Four-Dimensional Variational (4DVar), and the hybrid assimilation to further study the influence of all-sky GMI DA on typhoon analysis and prediction.

## Figures and Tables

**Table 1:** GPM microwave imager (GMI) sensor characteristics.

Channel	Frequency (GHz)	Polarization Mode	Scan Point (km)
1, 2	10.65	V, H	19.4 × 32.2
3, 4	18.7	V, H	11.2 × 18.3
5	23.8	V	9.2 × 15.0
6, 7	36.5	V, H	8.6 × 15.0
8, 9	89.0	V, H	4.4 × 7.3
10, 11	166	V, H	4.4 × 7.3
12	183 ± 3	V	4.4 × 7.3
13	183 ± 7	V	4.4 × 7.3

**Table 2:** Observation error, threshold of cloud liquid water path (CLWP) and observation standard deviation of GMI channels 5, 6 and 7.

Channel	Observation Error	Threshold of CLWP	Threshold of $\sigma_{0}$
5	1.60	0.25	8
6	1.18	0.10	6
7	2.67	0.10	6
